# Supplementary material for: Novel Driver Strength Index highlights important cancer genes in TCGA PanCanAtlas patients
Source: PeerJ. 2022 Aug 11;10:e13860. doi: 10.7717/peerj.13860 (PMC9375969; doi:10.7717/peerj.13860)
Supplement: Supplemental Information 2 [file peerj-10-13860-s002.zip › COHORTS/genes plots/cohorts/2021_8_16_14_9_distribution_cohorts_females_GBM.pdf]

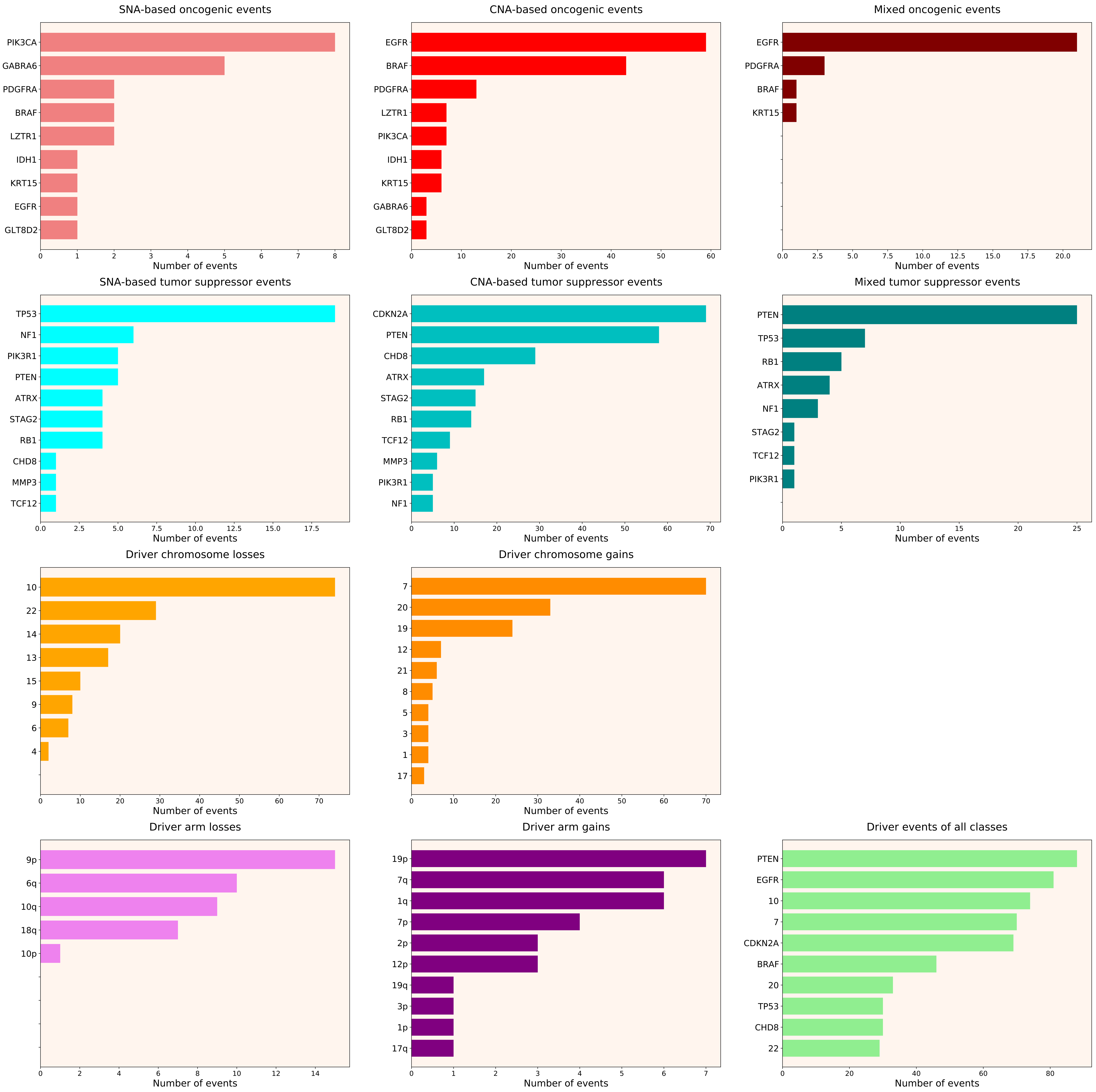
SNA-based oncogenic events
PIK3CA
GABRA6
PDGFRA
BRAF
LZTR1
IDH1
KRT15
EGFR
GLT8D2
0 1 2 3 4 5 6 7 8
Number of events
CNA-based oncogenic events
EGFR
BRAF
PDGFRA
LZTR1
PIK3CA
IDH1
KRT15
GABRA6
GLT8D2
0 10 20 30 40 50 60
Number of events
Mixed oncogenic events
EGFR
PDGFRA
BRAF
KRT15
0.0 2.5 5.0 7.5 10.0 12.5 15.0 17.5 20.0
Number of events
SNA-based tumor suppressor events
TP53
NF1
PIK3R1
PTEN
ATRX
STAG2
RB1
CHD8
MMP3
TCF12
0.0 2.5 5.0 7.5 10.0 12.5 15.0 17.5
Number of events
CNA-based tumor suppressor events
CDKN2A
PTEN
CHD8
ATRX
STAG2
RB1
TCF12
MMP3
PIK3R1
NF1
0 10 20 30 40 50 60 70
Number of events
Mixed tumor suppressor events
PTEN
TP53
RB1
ATRX
NF1
STAG2
TCF12
PIK3R1
0 5 10 15 20 25
Number of events
Driver chromosome losses
10
22
14
13
15
9
6
4
0 10 20 30 40 50 60 70
Number of events
Driver chromosome gains
7
20
19
12
21
8
5
3
1
17
0 10 20 30 40 50 60 70
Number of events
Driver arm losses
9p
6q
10q
18q
10p
0 2 4 6 8 10 12 14
Number of events
Driver arm gains
19p
7q
1q
7p
2p
12p
19q
3p
1p
17q
0 1 2 3 4 5 6 7
Number of events
Driver events of all classes
PTEN
EGFR
10
7
CDKN2A
BRAF
20
TP53
CHD8
22
0 20 40 60 80
Number of events
